# LY6K depletion modulates TGF‐β and EGF signaling

**DOI:** 10.1002/cam4.5940

**Published:** 2023-04-19

**Authors:** Sujeong Park, Doyeon Park, Sora Han, Ga Eun Chung, Sujung Soh, Hye In Ka, Hyun Jeong Joo, Young Yang

**Affiliations:** ^1^ Department of Biological Sciences Sookmyung Women's University Seoul Republic of Korea; ^2^ Novotech Seoul Republic of Korea; ^3^ Research Institute of Women's Health Sookmyung Women's University Seoul Republic of Korea

**Keywords:** cervical cancer, EGF, endocytosis, LY6K, TGF‐β

## Abstract

**Background:**

Lymphocyte antigen 6 complex locus K (LY6K), a glycosylphosphatidylinositol‐anchored protein, plays a dynamic role in cancer metastasis. In the current study, we deciphered the effects of LY6K on transforming growth factor‐β (TGF‐β) and epidermal growth factor (EGF) signaling through clathrin‐ and caveolin‐1 (CAV‐1)‐mediated endocytosis.

**Methods:**

Analysis of the TCGA and GTEx dataset were performed to explore the expression and survival of LY6K in cancer patients. Short interfering RNA (siRNA) was used to knockdown the expression of LY6K in human cervical cancer patients. The effect of lack of LY6K on cell proliferation, migration, and invasion was performed, and RT‐qPCR and immunoblotting were performed to identify LY6K‐affected TGF‐β and EGF signaling pathways. Additionally, Immunofluorescence (IF) and transmission electron microscope (TEM) were performed to identify the role of LY6K in CAV‐1‐ and Clathrin‐mediated endocytosis.

**Results:**

Lymphocyte antigen 6 complex locus K expression level is elevated in higher grade cervical cancer patients correlating with poor overall survival, progression‐free survival, and disease‐free survival. LY6K‐depletion in HeLa and SiHa cancer cells suppressed EGF‐induced proliferation and TGF‐β‐enhanced migration and invasion. Both TGF‐β receptor‐I (TβRI) and EGF receptor (EGFR) localized at the plasma membrane regardless of LY6K expression, and LY6K bound TβRI irrespective of the presence of TGF‐β; however, LY6K did not bind EGFR. LY6K‐depleted cells showed impaired Smad2 phosphorylation upon TGF‐β treatment and lower proliferation rates following long‐term treatment with EGF. We revealed the atypical movement of TβRI and EGFR from plasma membrane upon ligand stimulation in LY6K‐depleted cells and an impaired movement of the endocytic proteins clathrin and CAV‐1.

**Conclusions:**

Our study demonstrates the key role of LY6K in both clathrin‐ and CAV‐1‐mediated endocytic pathways regulated by TGF‐β and EGF, and it suggests a correlation between LY6K overexpression in cervical cancer cells and poor overall survival.

## Graphical abstract

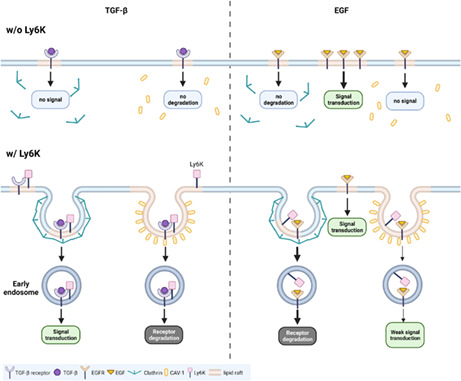

## 1 INTRODUCTION

Glycosylphosphatidylinositol (GPI)‐anchored lymphocyte antigen 6 complex locus K (LY6K) belongs to the Ly6/urokinase‐type plasminogen activator receptor family.^1^ LY6K is expressed in testicular cells and assists fertilization by facilitating the migration of spermatozoa toward the oviduct.^2^ Additionally, LY6K is also expressed in human cancer cells and is, therefore, one of the cancer/testis antigens (CTA). Its expression is normally restricted to the testicular tissues.^3^, ^4^ Since CTAs have a cancer‐restricted expression and immunogenic property, they are considered ideal for targeted immunotherapy.^5^, ^6^ Furthermore, LY6K localizes at the plasma membrane as a GPI‐anchored protein and affects various cellular functions, including cell differentiation, proliferation, migration, adhesion, and invasion in lung adenocarcinoma, esophageal cell carcinoma, breast cancer, and cervical cancer.^7^, ^8^, ^9^, ^10^ Enhanced expression level of LY6K correlates with poor overall survival (OS) in breast cancer, and LY6K induces the expression of programmed death‐ligand 1 (PD‐L1).^11^ LY6K has also been reported to suppress tumor immunity by increasing tumor‐infiltrating T regulatory cell numbers and decreasing natural killer (NK) cell activation.^11^ Furthermore, the deficiency of LY6K suppresses transforming growth factor‐ β (TGF‐β) signaling and thereby Smad1/5 and Smad2/3 signal transduction.^11^ Hypomethylation of LY6K promoter increased the expression levels of LY6K, and the increased expression level of LY6K results in poor OS in glioblastoma.^12^ LY6K binds caveolin‐1 (CAV‐1), and depletion of LY6K inhibits phosphorylation of the epidermal growth factor (EGF) activated extracellular signal‐regulated kinase (ERK); although the correlation of CAV‐1 with the regulation of EGF signaling pathway is still unclear.^12^ Therefore, it is likely that LY6K is involved in the regulation of TGF‐β and EGF signaling pathways, however, the molecular mechanisms underlying this regulation are not clearly understood yet.

Lipid rafts are highly ordered microdomains of the plasma membrane that are internalized via several distinct pathways.^13^, ^14^ Besides the well‐characterized clathrin‐dependent pathway, lipid rafts accommodating a GPI‐anchored protein are endocytosed via different mechanisms mediated by the classical clathrin‐coated pathway.^15^ They can also be endocytosed by caveolin‐dependent and caveolin‐independent endocytic pathways. Caveolae is a specialized form of lipid raft domain, the formation of which depends on the expression of CAV‐1.^16^ Interestingly, TGF‐β receptor internalization can occur via two pathways (clathrin‐dependent and caveolae‐dependent).^17^, ^18^ Upon TGF‐β treatment, CAV‐1‐mediated TGF‐β receptor internalization induces receptor degradation, leading to an abrogated TGF‐β signal.^19^ Whereas clathrin‐mediated TGF‐β receptor endocytosis activates Smad signaling pathway to promote cancer metastasis.^20^ Furthermore, clathrin‐mediated EGF receptor (EGFR) endocytosis induces receptor degradation, dampening the EGF signal.^21^ EGF and EGFR binding activates AKT and ERK signaling at the plasma membrane.^22^, ^23^ However, the factors that affect these pathway and whether these pathways are interconnected are still not completely understood.

In this study, we explored the effects of LY6K on TGF‐β and EGF signaling pathways in cervical cancer cells. In the absence of LY6K, impaired clathrin‐mediated endocytosis reduced TGF‐β signaling, while it enhanced EGF signaling in a short‐term period (1 h) due to an attenuated signal. Additionally, LY6K depletion suppressed CAV‐1‐mediated endocytosis. LY6K critically regulates both clathrin‐ and CAV‐1‐mediated pathways of endocytosis.

## 2 MATERIALS AND METHODS

### 2.1 Cell culture

HeLa, SiHa, and HEK 293T cells were cultured in Dulbecco's modified Eagle medium (DMEM; Cytiva) supplemented with 10% fetal bovine serum (FBS; Gibco) at 37°C in 5% CO₂ humidified atmosphere. MDA‐MB‐468, U‐937, and A549 cells were cultured in Roswell Park Memorial Institute (RPMI)‐1640 medium (HyClone) supplemented with 10% FBS under same conditions.

### 2.2 Reagents and antibodies

The following primary antibodies were used for immunoblotting: mouse monoclonal anti‐LY6K (Santa Cruz Biotechnology; sc‐393560), sheep polyclonal anti‐LY6K (R&D Systems; AF6648), rabbit monoclonal anti‐caveolin‐1 (Cell Signaling Technology; #3267), rabbit polyclonal anti‐TGFβRI (Santa Cruz Biotechnology, sc‐398), rabbit polyclonal anti‐TGFβRI (Santa Cruz Biotechnology, sc‐402), rabbit monoclonal anti‐EGF receptor (Cell signaling Technology, #4267), rabbit monoclonal anti‐clathrin heavy chain (Cell Signaling Technology, #4796), goat polyclonal anti‐Smad2/3 (Santa Cruz Biotechnology, sc‐6033), rabbit polyclonal anti‐pSmad2 (Ser465/467) (Cell Signaling Technology, #3101), rabbit monoclonal anti‐pSmad3 (Ser423/425) (Cell Signaling Technology, #9520), rabbit monoclonal anti‐AKT (pan) (Cell Signaling Technology, #4691), rabbit monoclonal anti‐pAKT (Ser473) (Cell Signaling Technology, #4060), rabbit polyclonal anti‐p44/42 MAPK (ERK1/2) (Cell Signaling Technology, #9102), rabbit polyclonal anti‐p44/42 MAPK (ERK1/2) (Cell Signaling Technology, #9102), mouse monoclonal anti‐pp44/42 MAPK (ERK1/2) (Thr202/Tyr204) (Cell signaling Technology, #9106). The following primary antibodies were used for immunoprecipitation: mouse monoclonal anti‐LY6K (Santa Cruz Biotechnology, sc‐393560), rabbit polyclonal anti‐TGFβRI (Santa Cruz Biotechnology, sc‐398), rabbit monoclonal anti‐EGF receptor (Cell signaling Technology, #4267), mouse IgG isotype control (Santa Cruz Biotechnology, sc‐2025). The following primary antibodies were used for immunofluorescence: mouse monoclonal anti‐LY6K (Santa Cruz Biotechnology, sc‐393560), rabbit polyclonal anti‐TGFβRI (Santa Cruz Biotechnology, sc‐398), rabbit monoclonal anti‐EGF receptor (Cell signaling Technology, #4267), rabbit monoclonal anti‐EEA1 (C45B10) (Cell Signaling Technology, #3288), rabbit monoclonal anti‐caveolin‐1 (Cell Signaling Technology, #3267), rabbit monoclonal anti‐clathrin heavy chain (Cell Signaling Technology, #4796). HRP‐conjugated goat anti‐mouse or anti‐rabbit IgG (Fab) secondary antibodies were purchased from Enzo Life Sciences, HRP‐conjugated mouse anti‐goat IgG from Santa Cruz Biotechnology, and HRP‐conjugated donkey anti‐sheep from R&D Systems. Recombinant human TGF‐β1 protein was purchased from R&D Systems.

### 2.3 Plasmids and RNA interference

Human TGFβR1 cloned in mCherry‐N1 vector (#54969) was purchased from Addgene. pCMV6‐LY6K was a gift from Prof. Jong Hoon Park, Sookmyung Women's University. Plasmid transfections were performed using Jetprime (Polyplus‐transfection) reagent according to the manufacturer's protocol. To silence LY6K expression, cells were transfected with 20 ng/L siRNA using Lipofectamine RNAiMAX transfection reagent (Invitrogen). siRNAs were synthesized by Bioneer with sequence: 5′‐UUUCUCUCUCACAAACAUG‐3′ (siLY6K #2). 5′‐AAGGAGGUGCAAAUGGACAGA‐3′ (siLY6K #3).

### 2.4 Reverse transcription quantitative polymerase chain reaction

Total RNA was isolated using RNAiso Plus (TaKaRa), where 500 μL of the reagent was added to each cell culture in a 6‐well plate. The cell suspensions were mixed with 100 μL chloroform in Eppendorf tubes, centrifuged at 18,000 × g for 15 min at 4°C, and 200 μL supernatant was harvested. It was then mixed with an equal volume of isopropanol and centrifuged at 18,000 × g for 15 min at 4°C. The pellets were dissolved in ultrapure water (Invitrogen). The extracted RNA was reverse transcribed using RevertAid RT Reverse Transcription Kit (Thermo Fisher Scientific), and RT‐qPCR was performed using 2× Q‐PCR Master Mix (SMOBIO) and LY6K specific primers (forward: 5′‐AGCCCATGCCCTTCTTTTACCTCA‐3′; reverse: 5′‐CCAGCCACAGCCCACAG‐3′). The expression levels were normalized with β‐actin level.

### 2.5 Immunoblotting

Cells were lysed using GST ‐IP (50 mM Tris–HCl pH 8.0, 150 mM NaCl, 5 mM EDTA, 1% (v/v) NP‐40, and a protease inhibitor cocktail tablet [Roche]) and incubated for 15 min on ice. After centrifugation at 18,000 × g, the supernatant was mixed with 5× SDS and boiled at 99°C for 10 min. The proteins were separated by SDS polyacrylamide gel electrophoresis and transferred onto a 0.45 μm nitrocellulose membrane (Cytiva). The membranes were blocked with 3% (w/v) BSA in TBS‐T for 20 min and incubated overnight with the appropriate primary antibodies at 4°C. The membranes were then washed with TBS‐T and incubated with HRP‐conjugated secondary antibodies in 5% (w/v) skim milk prepared in TBS‐T at room temperature. The protein signal was detected with the ECL solution and analyzed in a Fusion Solo‐S image analyzer (Vilber Lourmat).

### 2.6 Proliferation and wound healing assay

HeLa and SiHa cells were seeded into 96‐well plates after transfection with siRNA. The cells were photographed using a Lionheart FX Automated Microscope (Agilent) at 37°C/5% CO_2_ for 48 h. The cell area was calculated using the Gen5 software.

For the wound healing assay, cells were seeded into 6‐well plates. After 48 h post‐transfection, the cell monolayer was manually wounded with a 200 μL micropipette tip. The cells were imaged using Lionheart FX Automated Microscope (Agilent) at 37°C/5% CO₂ for 48 h. The wound closure was calculated using the Gen5 software.

### 2.7 Invasion assay

The cells were seeded into the upper chamber of inserts (8 μm pore size, 6.5 mm insert; SPL) coated with Matrigel (Corning). After 24 h, the cells and medium were removed from the upper chamber and washed with PBS. The invasive cells were then fixed by placing the inserts into 4% paraformaldehyde in a 24‐well plate. After drying the inserts, 1% crystal violet (Sigma‐Aldrich) was added to the 24‐well plate to stain the cells. The inserts were then carefully washed with distilled water and dried using a cotton swab.

### 2.8 Immunoprecipitation

Cells were lysed with the lysis buffer (50 mM Tris HCl pH 7.5, 100 mM NaCl, 1% v/v TritonX‐100, 1 mM EDTA, and 10% v/v glycerol). Following centrifugation, 1 mg cell lysate protein was incubated with 1 μg antibody for 2 h at 25°C and then with 30 μL protein G beads (Amicogen) overnight at 4°C. The immunocomplexes were washed (4 times) with lysis buffer and mixed with 5× SDS buffer to perform immunoblotting as described above (Section 2.5).

### 2.9 Endosome isolation

Endosome compartments were isolated from HeLa and SiHa cells using a Minute™ Endosome Isolation and Cell Fractionation Kit (Invent Biotechnologies; ED‐028) as indicated. Briefly, cells were resuspended in buffer A and incubated for 10 min. The filter cartridge filled with cell extracts was centrifuged and the pellet was obtained as a plasma membrane. The supernatant added buffer B was incubated overnight at 4°C. The endosome pellet was resuspended with RIPA buffer.

### 2.10 Immunofluorescence

HeLa and SiHa cells were cultured onto glass coverslips and fixed with 4% (v/v) formaldehyde (Sigma‐Aldrich) diluted in PBS for 15 min at room temperature. The cells were then washed with PBS, permeabilized, and blocked with 0.1% Triton X‐100 and 3% BSA in PBS for 15 min at room temperature. The cells fixed onto coverslips were incubated with the primary antibody at 4°C overnight and then with secondary antibody at room temperature for 2 h. DAPI (Sigma‐Aldrich) was utilized for staining nuclei.

### 2.11 Transmission electron microscopy

Cells were fixed with 2% paraformaldehyde and 2.5% (v/v) glutaraldehyde (Sigma‐Aldrich) in 0.1 M phosphate buffer pH 7.2 at 4°C overnight. The fixed cells were washed with 0.05 M sodium cacodylate buffer, postfixed with osmium tetroxide (OsO4) at 4°C for 1 h, and then stained with 0.5% uranyl acetate in distilled water at 4°C overnight. The samples were serially dehydrated with increasing concentrations of ethanol (30%, 50%, 70%, 80%, 90%, and 100%) and embedded in Spurr's resin. For TEM imaging, ultrathin sections (70 nm) of solid resin were prepared using an ultramicrotome (UC7; Leica), and the specimens were photographed under a transmission electron microscope (LIBRA 120; Carl Zeiss).

### 2.12 Bioinformatic analysis

LY6K mRNA expression levels in human cervical cancer patients and healthy individuals were obtained from the UCSC Xena Project (http://xena.ucsc.edu/) while its expression levels at the cervical cancer stage and survival outcomes were obtained from cBioportal (https://www.cbioportal.org/). Its expression levels during human cervical cancer progression from normal to grade 3 stage were obtained from GSE63514. LY6K mRNA expression in various cancer patients was acquired from GEPIA (http://gepia.cancer‐pku.cn/).

### 2.13 Statistical analysis

Values are presented as mean ± standard deviation (SD). One‐way ANOVA and Two‐way ANOVA were used for multiple comparisons within groups, and individual group mean differences were analyzed using two‐tailed Student's *t*‐test. Values of *p < 0.05, **p < 0.01, and ***p < 0.001 were considered benchmarks of significant differences. All statistical analyses were performed using GraphPad Prism 8.0.

## 3 RESULTS

### 3.1 Expression level of LY6K is elevated in cervical cancer

**FIGURE 1:**
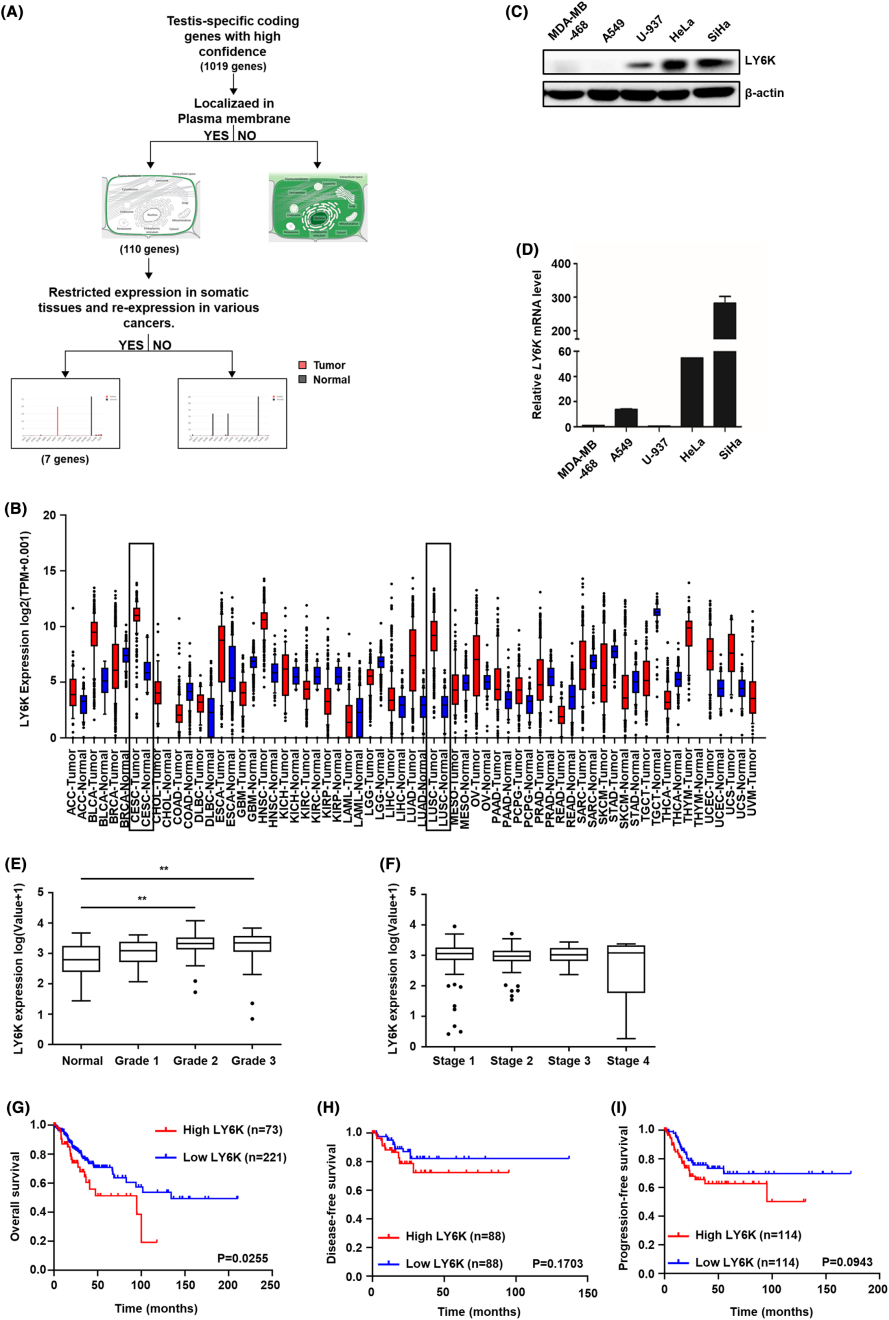
Cervical cancer patients show high lymphocyte antigen 6 complex locus K (LY6K) expression and poor overall survival. (A) Flow chart of gene selection. (B) RNA‐seq analysis of LY6K in normal and tumor cells. (C) Immunoblot analysis of LY6K expression. (D) LY6K mRNA level analyzed by RT‐qPCR. (E) LY6K expression at different grades of cervical cancer; **p < 0.01 by one‐way ANOVA with Tukey's test. (F) LY6K expression during different stages of cervical cancer. (G–I) Patients with high and low levels of LY6K expression were classified, and the percentage of overall survival (G), disease‐free survival (H), and progression‐free survival (I) was acquired from cBioportal TCGA dataset. CESC, cervical squamous cell carcinoma and endocervical adenocarcinoma; LUSC, lung squamous cell carcinoma.

The plasma membrane‐localized CTA was selected from 110 genes encoding membrane‐localized proteins, obtained after GeneCards database analysis (http://www.genecards.org) of 1019 CTA gene candidates previously published as highly expressed CTA genes.^24^ The expression levels of these 110 genes were compared among various human cancer and normal tissues (http://gepia.cancer‐pku.cn/index.html). The workflow has been presented diagrammatically in Figure 1A. Finally, seven genes showing high expressional variation between normal and cancerous tissues were selected. Among these, LY6K was significantly upregulated in lung and cervical cancer tissues than in others (Figure 1B). Indeed, high LY6K expression was observed in human cervical cancer cells viz. HeLa and SiHa cells (Figure 1C). Although LY6K mRNA expression levels were lower in the U937 cells than in HeLa and SiHa cells (Figure 1D), LY6K protein expression was detected, suggesting translational regulation. Furthermore, the Cancer Genome Atlas (TCGA) database and Gene Expression Omnibus (GEO) cohort were used to analyze LY6K mRNA expression in association with the pathological characteristics, where LY6K was upregulated during higher grade in cervical cancer patients (Figure 1E). However, the LY6K expression level showed no significant difference between the cervical cancer metastatic stages in the TCGA database (Figure 1F). Cervical cancer patients with high levels of LY6K expression showed poor OS. Although the difference in both disease‐free and progression‐free survival rates was not statistically significant, they exhibited a tendency toward poor outcomes (Figure 1G–I).

### 3.2 Loss of LY6K inhibits proliferation, migration, and invasiveness of cervical cancer cells

**FIGURE 2:**
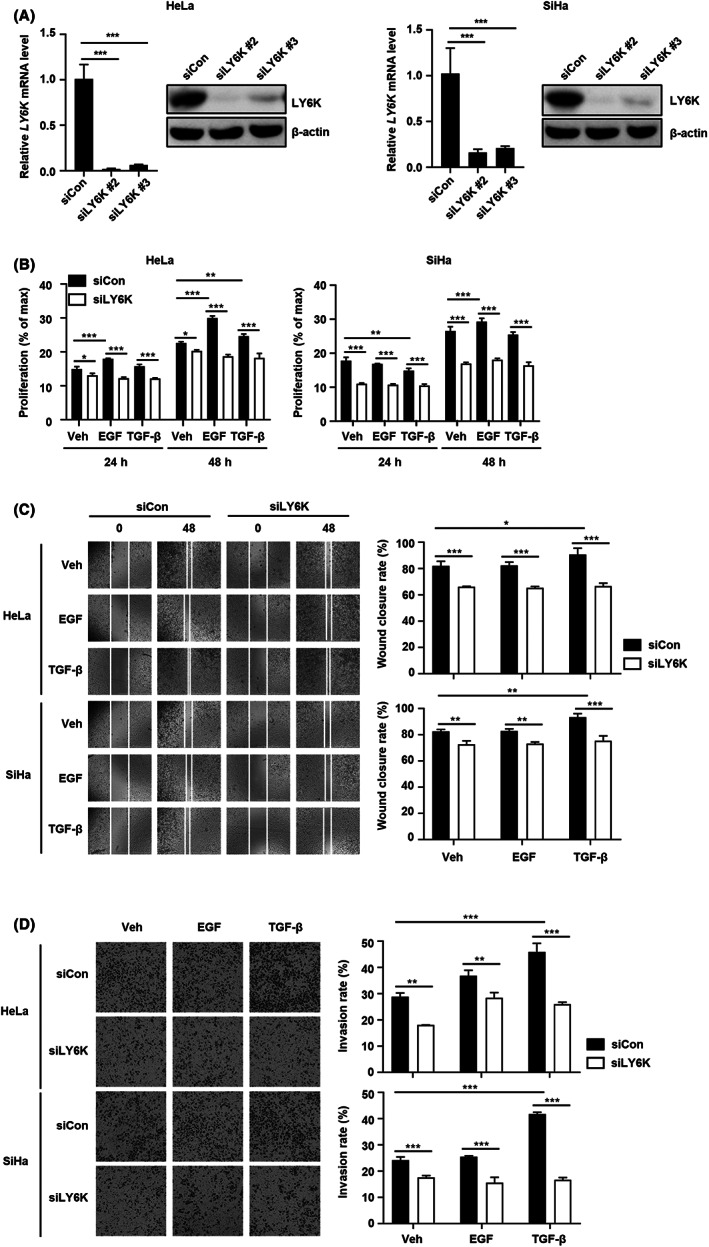
Depletion of lymphocyte antigen 6 complex locus K (LY6K) suppresses proliferation, migration, and invasiveness of cervical cancer cells. (A) HeLa and SiHa cells were transfected with 20 ng/L of siLY6K for 48 h, protein and mRNA level of LY6K were then determined; ***p < 0.001, by one‐way ANOVA with Tukey's test. (B) Proliferation of HeLa and SiHa cells transfected with siLY6K measured at 24 and 48 h (*n* = 3); ***p < 0.001, **p < 0.01, *p < 0.05, by two‐way ANOVA with Tukey's test. (C) The siLY6K‐transfected cells were scratched and monitored for wound closure after 48 h. Representative images of *n* = 3 are shown; ***p < 0.001, **p < 0.01, *p < 0.05 by two‐way ANOVA with Tukey's test (scale bar, 100 μm). (D) The siLY6K‐transfected cells at 24 h post‐incubation, the invasive cells were stained with crystal violet and photographed. Representative images of *n* = 3 are shown; ***p < 0.001, **p < 0.01 by two‐way ANOVA with Tukey's test (scale bar, 100 μm).

The depletion of LY6K in breast cancer cells decreases their migration and invasion^7^; therefore, the effect of LY6K depletion in cervical cancer cells was examined on their proliferation, migration, and invasion. HeLa and SiHa cells were transfected with two siLY6Ks and the loss of LY6K expression was confirmed by RT‐PCR and immunoblot assays 48 h post‐transfection (Figure 2A). The selected siLY6K #2 was used for subsequent experiments. Inhibition in the basal level of proliferation was observed upon LY6K depletion in these cells, and the increase in proliferation exhibited upon EGF treatment was abolished. Additionally, the impairment of EGF‐induced proliferation was greater than that observed upon TGF‐β induction in both HeLa and SiHa cells (Figure 2B). Furthermore, the native cells showed higher migration and invasion rates 48 h post‐treatment with TGF‐β, and these effects were abolished upon LY6K depletion. Such impaired migration and invasiveness in the LY6K‐depleted HeLa and SiHa cells was larger upon induction with TGF‐β than with EGF (Figure 2C,D). Therefore, LY6K depletion suppresses the effects of EGF and TGF‐β along with the basal levels of proliferation, migration, and invasiveness in cervical cancer cells. These results indicate the role of LY6K in the proliferation, migration, and invasiveness of cervical cancer cells, which enhance upon treatment with TGF‐β and EGF.

### 3.3 Depletion of LY6K reduces TGF‐β‐mediated Smad activation but increases EGF signaling pathway

**FIGURE 3:**
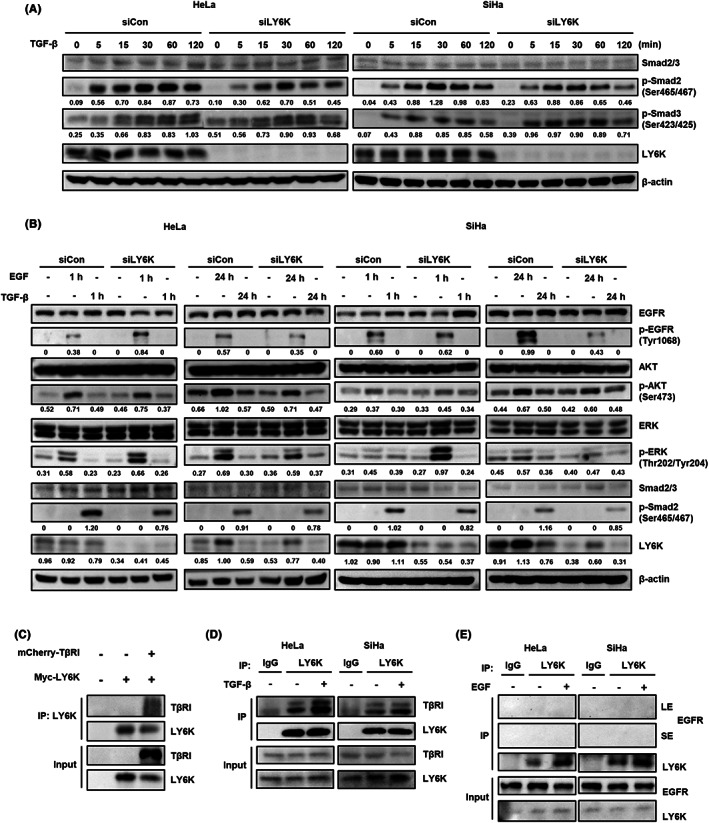
Lymphocyte antigen 6 complex locus K (LY6K) binds TGF‐β receptor‐I (TβRI) but not EGF receptor (EGFR) and regulates transforming growth factor‐β (TGF‐β) and epidermal growth factor (EGF) signaling pathways. (A) Time course of changes in Smad signaling in LY6K‐depleted cells. The intensities of p‐Smad2 and 3 band were quantified with ImageJ software. (B) Cells transfected with 20 ng/L of siLY6K for 48 h were incubated with 5 ng/mL of TGF‐β and 10 ng/mL of EGF for 1 and 24 h. The intensities of p‐EGFR, p‐AKT, p‐ERK, p‐Smad2, and LY6K band were quantified with ImageJ software. (C) HEK293T cells were transfected with Myc‐LY6K or/and mCherry‐TβRI and LY6K immunoprecipitated with anti‐LY6K antibody; levels of LY6K and TβRI were examined. (D, E) HeLa and SiHa cells were incubated with 5 ng/mL of TGF‐β (D) and 10 ng/mL of EGF (E) for 1 h, and LY6K immunoprecipitated with anti‐LY6K antibody. LE, long exposure; SE, short exposure.

Since TGF‐β‐ and EGF‐induced proliferation, migration, and invasiveness are affected in LY6K‐depleted cervical cancer cells, the effect of TGF‐β and EGF on signaling pathways was further studied in such cells. TGF‐β‐induced Smad2 phosphorylation was significantly suppressed in LY6K‐depleted HeLa and SiHa cells. However, Smad3 phosphorylation showed no appreciable alteration (Figure 3A). LY6K‐depleted HeLa and SiHa cells also showed enhanced phosphorylation of EGFR and ERK 1 h after EGF treatment (Figure 3B). Since the proliferation propensity of LY6K‐depleted cervical cancer cells is low, the enhanced EGF signaling pathway was further resolved. The phosphorylation of EGFR, AKT, and ERK was examined 1‐day post‐treatment with EGF rather than short‐term treatment. The EGF signaling pathway was attenuated upon LY6K depletion, whereas no variations were observed in the TGF‐β signaling pathway between short‐term and long‐term treatments with TGF‐β (Figure 3B). To rule out potential off‐target effect of the siRNA, we examined the TGF‐β and EGF signaling pathways using another siLY6K#3. However, no significant differences were observed, indicating that the observed effect were specific to LY6K depletion (Figure S1).

Furthermore, the TGF‐β signaling pathway is altered in LY6K‐depleted cells; therefore, the interaction between LY6K and TGF‐β receptor‐I (TβRI) at the plasma membrane was assessed. Myc‐tagged LY6K and mCherry‐tagged TβRI were highly expressed in HEK293T cells. The LY6K was pulled down, where it co‐immunoprecipitated with TβRI (Figure 3C). To further confirm their interaction, HeLa and SiHa cells were treated with TGF‐β, and lysates were immunoprecipitated with anti‐LY6K antibody to examine endogenous interaction. LY6K was observed to bind TβRI, regardless of the presence of TGF‐β (Figure 3D). In contrast to TβRI, EGFR did not bind endogenous LY6K in HeLa and SiHa cells regardless of the presence of EGF (Figure 3E). Conclusively, LY6K‐depletion associates with the inhibition and activation of TGF‐β and EGF signaling pathways, respectively, during their short‐term treatments.

### 3.4 Depletion of LY6K suppresses TβRI and EGFR‐mediated endocytosis

**FIGURE 4:**
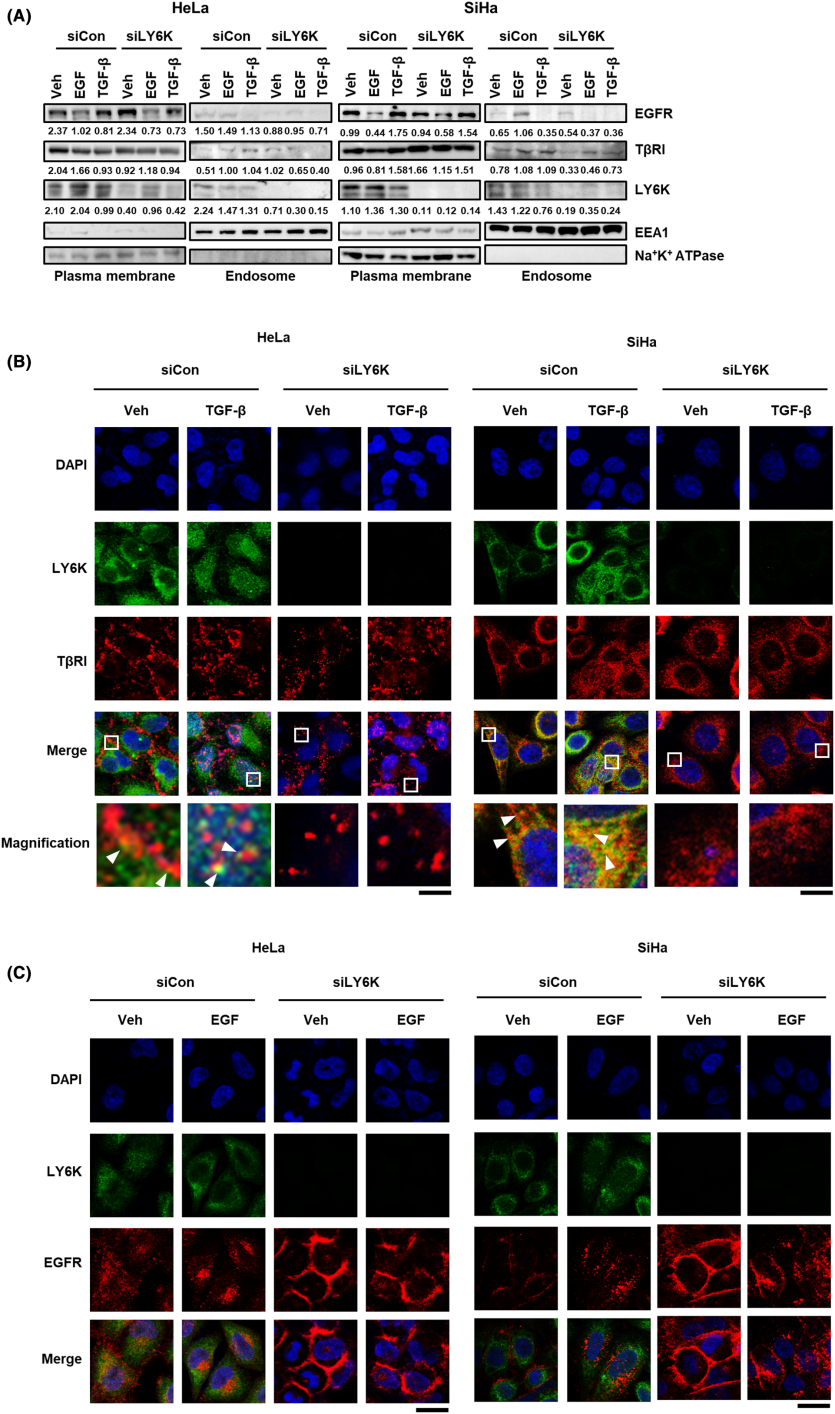
Depletion of lymphocyte antigen 6 complex locus K (LY6K) inhibits TGF‐β receptor‐I (TβRI) and EGF receptor (EGFR) trafficking from plasma membrane to endosome. (A) HeLa and SiHa cells transfected with 20 ng/L of siLY6K were treated with 5 ng/mL of transforming growth factor‐β (TGF‐β) and 10 ng/mL of epidermal growth factor (EGF) for 1 h. The plasma membrane, endosome and cytosolic fractions were evaluated using Na+/K+ ATPase, EEA1 and α‐tubulin as respective markers. (B, C) The siLY6K‐transfected cells were treated with 5 ng/mL of TGF‐β or 10 ng/mL of EGF for 1 h and immuno‐stained for endogenous LY6K, TβRI (B), and EGFR (C). DAPI was used to stain nuclei (scale bar, 20 μm).

The co‐localization of TβRI and EGFR with LY6K in the plasma membrane and endosome was examined to explore the underlying mechanism by which LY6K affects TGF‐β and EGF signaling pathways. After the treatment of TGF‐β and EGF, the levels of TβRI and EGFR were examined at the plasma membrane and endosome. In LY6K‐depleted cells, treatment of TGF‐β and EGF resulted in reduced levels of TβRI and EGFR in endosome (Figure 4A), suggesting that endocytosis of TβRI and EGFR is mediated by LY6K. To further confirm, the localization of TβRI and EGFR was examined using immunofluorescence. Following treatment with TGF‐β, TβRI and LY6K predominantly colocalized at the plasma membrane, while a fraction of them migrated to the endosome in HeLa and SiHa cells (Figure 4B). However, in the absence of LY6K, TβRI scattered at the plasma membrane, and TGF‐β treatment did not affect its migration into the endosome from the plasma membrane (Figure 4B).

Contrastingly, LY6K did not colocalize with EGFR, and EGF treatment promoted endosome localization of EGFR; however, upon LY6K depletion, EGFR remained at the plasma membrane (Figure 4C). These results indicate that TβRI and EGFR localize at the plasma membrane, and receptor‐mediated endocytosis in LY6K‐depleted cells rarely occurs in response to TGF‐β and EGF, suggesting the implication of LY6K in TGF‐β‐ and EGF‐mediated signaling pathways.

### 3.5 Depletion of LY6K decreases Clathrin‐ and CAV‐1‐mediated endocytosis

**FIGURE 5:**
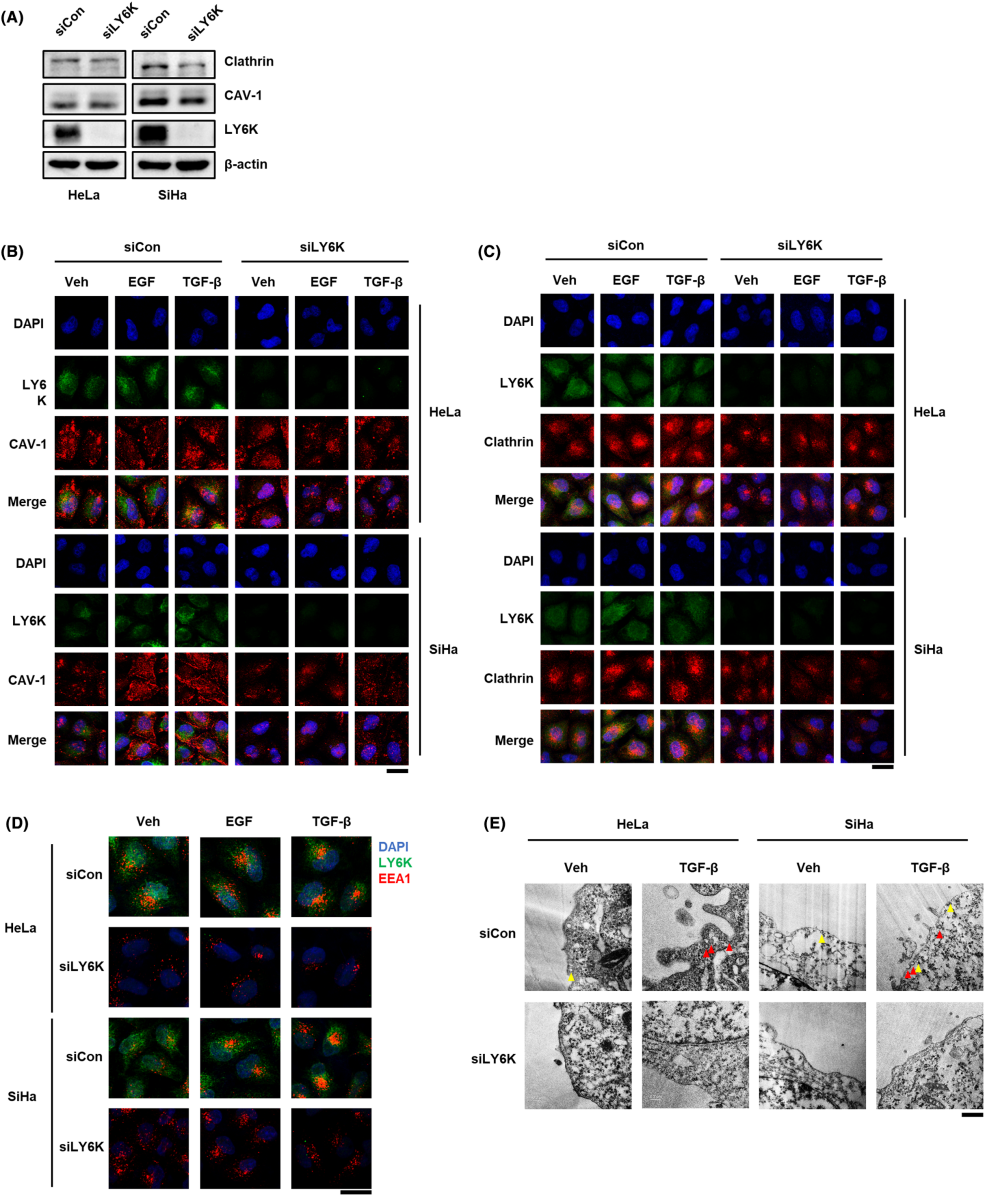
Depletion of lymphocyte antigen 6 complex locus K (LY6K) negatively regulates clathrin‐ and CAV‐1‐mediated endocytic pathways. (A) Cells transfected with 20 ng/L of siLY6K for 48 h were used to examine clathrin and CAV‐1 levels. (B, D) The siLY6K‐transfected cells were treated with 5 ng/mL of transforming growth factor‐β (TGF‐β) and 10 ng/mL of epidermal growth factor (EGF) for 1 h and immuno‐stained for endogenous LY6K, CAV‐1 (B), clathrin (C), EEA1 (D). DAPI was used to stain nuclei. (scale bar, 20 μm) (E) HeLa and SiHa cells transfected with siLY6K for 48 h were incubated with 5 ng/mL of TGF‐β. The ultrastructure of caveolar vesicles and clathrin‐coated vesicles was captured by transmission electron microscope (TEM); clathrin‐mediated endocytosis: red arrows; CAV‐1‐mediated endocytosis: yellow arrows (scale bar, 500 nm).

LY6K is involved in clathrin‐ and CAV‐1‐mediated signal transfer, and the signals of EGF and TGF‐β are transferred by receptor endocytosis at the plasma membrane,^25^, ^26^ therefore, alterations in the endocytic proteins, clathrin and CAV‐1, as an effect of LY6K depletion were examined. In the absence of LY6K, the clathrin levels decreased in both HeLa and SiHa cells, but CAV‐1 levels only decreased in SiHa cells (Figure 5A). Furthermore, the localization of clathrin and CAV‐1 was examined in the presence and absence of LY6K. CAV‐1 clustered in the lipid rafts in both HeLa and SiHa cells and migrated into the endosome in response to TGF‐β but not EGF treatment in HeLa cells, while in response to both in SiHa cells. Although HeLa and SiHa cells are cervical cancer cell lines, they are different types of cancer. HeLa is an adenocarcinoma cell line, while SiHa is a squamous cell carcinoma cell line. Although there is a report suggesting differential performance of CAV‐1 among breast cancer cell lines,^27^ the underlying reasons for this variation are yet to be elucidated. However, no such cytosolic migration was observed upon LY6K depletion, despite TGF‐β and EGF treatment (Figure 5B), indicating that LY6K is required during CAV‐1‐mediated endocytosis. Concerning clathrin, both LY6K‐depleted HeLa and SiHa cells showed lower clathrin levels in the plasma membrane and less perinuclear clustering compared with non‐depleted cells. Even after treatment with TGF‐β and EGF, the localization of clathrin remained unaltered in absence of LY6K (Figure 5C), indicating that clathrin‐mediated endocytosis reduces in the absence of LY6K.

The proteins endocytosed via clathrin and CAV‐1 are delivered to early endosome antigen‐1 (EEA1)‐positive endosomes.^28^, ^29^ The early endosomes undergo maturation and move to the perinuclear region after forming clusters.^30^ Both HeLa and SiHa cells displayed scattered localization of EEA1‐positive endosomes throughout cytosol, which moved to the perinuclear region following EGF and TGF‐β treatment for 1 h. However, this perinuclear movement in response to EGF and TGF‐β was impaired upon LY6K depletion (Figure 5D).

The involvement of LY6K in TGF‐β‐mediated endocytosis was further confirmed where endocytic compartments were observed by transmission electron microscopy. CAV‐1‐mediated endocytosis regulated TβRI levels, but clathrin‐mediated endocytosis did not occur in the absence of TGF‐β. However, in presence of TGF‐β, clathrin‐mediated endocytosis was high in both HeLa and SiHa cells, whereas it was impaired following LY6K depletion (Figure 5E). Conclusively, LY6K plays an important role in membrane endocytosis of cancerous cells, irrespective of the type of endocytosis.

## 4 DISCUSSION

**FIGURE 6:**
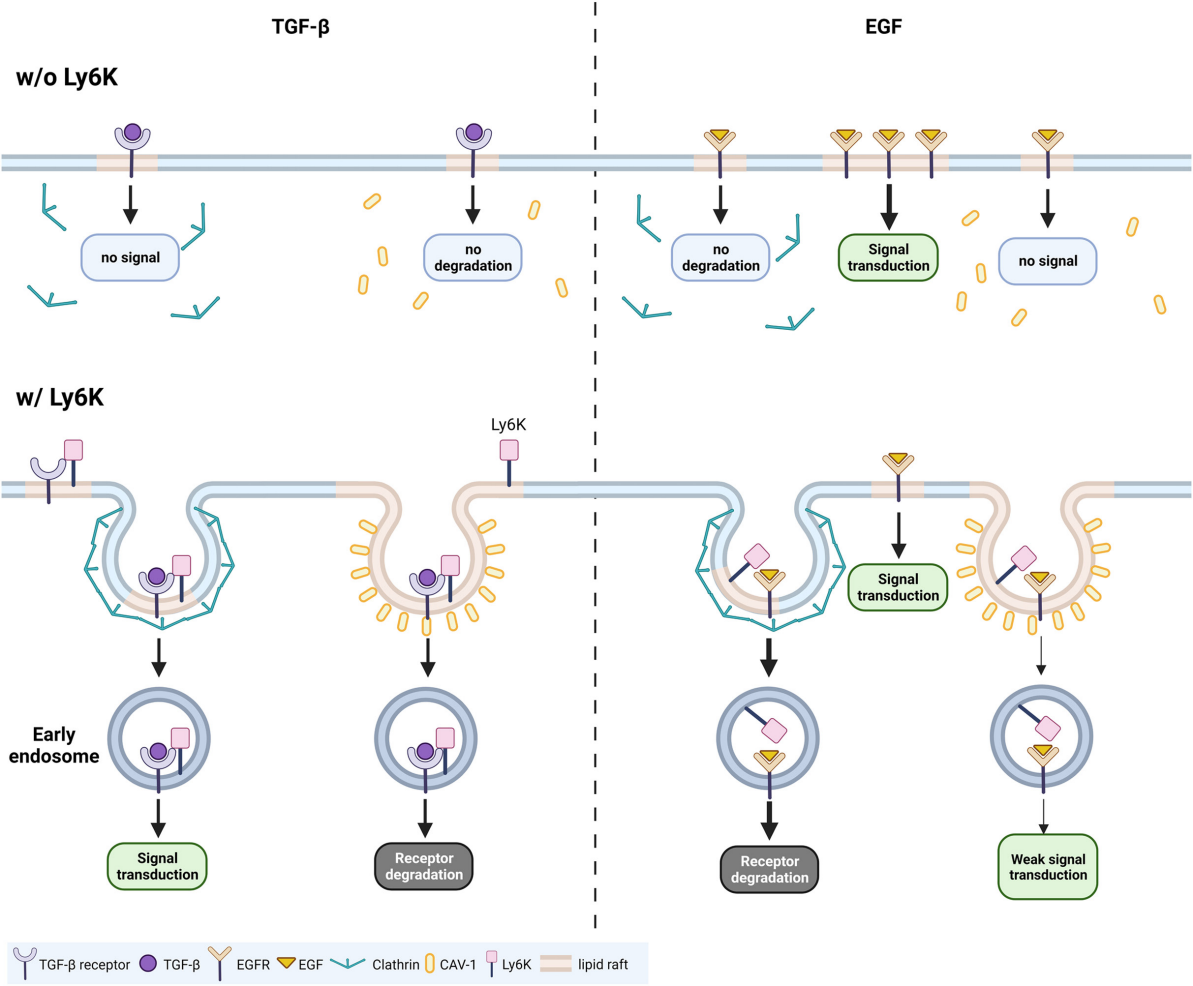
Schematic representation of lymphocyte antigen 6 complex locus K (LY6K)‐mediated regulation of transforming growth factor‐β (TGF‐β) and epidermal growth factor (EGF) signaling pathways. Their receptors are internalized via clathrin and CAV‐1‐mediated endocytosis, and the vesicles fuse with early endosome antigen‐1 (EEA1) positive endosomes. LY6K arbitrates clathrin‐ and CAV‐1‐mediated endocytosis of TGF‐β and EGF receptors, thereby regulating these signaling pathways. The clathrin‐mediated endocytosis of TGF‐β receptor stimulates signal transduction, while CAV‐1‐mediated endocytosis induces receptor degradation, suppressing TGF‐β signaling. In the absence of LY6K, no signal transduction occurs due to inhibited clathrin‐mediated endocytosis. Signal transduction mainly occurs at the plasma membrane during EGF signaling. The clathrin‐mediated endocytosis of EGF receptor (EGFR) induces degradation for receptor desensitization. However, in the absence of LY6K, no EGFR degradation occurs due to impaired clathrin‐mediated endocytosis, which thereby activates the signaling pathway. Created with BioRender.com.

The activation of TGF‐β and EGF signaling pathways plays a critical role in cancer metastasis and proliferation, respectively, where LY6K is required for TGF‐β‐ and EGF‐induced metastasis in breast cancer and glioblastoma.^11^, ^12^ However, little is known concerning molecular mechanisms by which LY6K affects TGF‐β‐ and EGF‐induced metastasis at the receptor level. In this study, we report that LY6K‐depletion suppresses the effect of TGF‐β on invasiveness and EGF‐stimulated proliferation of tumor cells by repressing clathrin‐ and CAV‐1‐mediated endocytosis (Figure 6).

In higher‐grade cervical cancer patients, elevated LY6K expression level was observed correlating with poor prognosis. Additionally, patients with high LY6K expression level showed poor overall, progression‐free, and disease‐free survival. Since LY6K is a GPI‐anchored protein and its depletion downregulates the proliferation, migration, and invasion of cervical cancer cells, the signal transduction mechanisms were explored at surface receptor level. Endogenous LY6K directly bound TβRI, and its depletion suppressed TGF‐β‐induced Smad activation. Therefore, LY6K is associated with TGF‐β‐mediated signaling pathway. Contrastingly, LY6K‐depleted HeLa and SiHa cells showed increased AKT and ERK activation during short‐term EGF treatment but decreased proliferation upon long‐term treatments. However, endogenous LY6K did not bind EGFR. Since LY6K depletion affected downstream signals of TGF‐β and EGF, including invasiveness and proliferation of cervical cancer cells, it is possible that LY6K is an important regulator of signal transduction of ligands at the plasma membrane level. Moreover, LY6K depletion inhibited basal levels of proliferation and invasiveness, suggesting its involvement in basal level receptor recycling. However, the association of LY6K with other functions of TGF‐β and EGF beyond cell proliferation and wound healing remains unclear.

Receptor‐ligand binding upon TGF‐β and EGF induction triggers internalization of the complex for appropriate signal transduction or receptor desensitization.^17^, ^31^, ^32^ Although LY6K is involved in the regulation of EGF and TGF‐β signaling pathways, it binds TβRI but not EGFR indicating its involvement in membrane endocytosis, at least in case of EGFR. LY6K depletion enhanced EGFR levels at plasma membrane leading to a short‐term increase in EGF downstream signal. EGFR is degraded via clathrin‐dependent endocytosis^22^, ^33^; therefore, impaired clathrin‐mediated endocytosis in LY6K‐depleted cells resulted in membrane retention of EGFR followed by an increased downstream signal. However, long‐term EGF treatment suppressed proliferation rather than increasing it, and failure of EGFR recycling during long‐term periods is likely to abrogate the EGF signal. Furthermore, depletion of LY6K also increased TβRI levels at the plasma membrane. In contrast to EGF, clathrin‐dependent endocytosis activates TGF‐β signal^20^, ^34^; consequently, TGF‐β signal is inhibited in the LY6K‐depleted cells. Moreover, regardless of LY6K binding to EGFR and TβRI, its depletion impaired receptor‐mediated endocytosis. Therefore, LY6K can critically affect endocytosis by regulating the invagination of its membrane anchoring regions, regardless of receptor binding.

Following signal induction, the receptors are mostly internalized via clathrin‐ and CAV‐1‐mediated endocytosis, effecting the receptor‐specific signaling pathway.^17^, ^35^ TGF‐β signaling is mainly activated by clathrin‐mediated endocytosis but inhibited by CAV‐1‐dependent endocytosis.^17^ EGF signaling, which is mainly activated at plasma membrane, is inhibited by clathrin‐mediated endocytosis, preventing continuous oncogenic signaling.^22^ However, CAV‐1‐dependent endocytosis marginally transduces the EGF signal.^36^, ^37^ Following endocytosis, the clathrin‐mediated endocytic vesicles fuse with EEA1 positive endosomes.^38^ Subsequently, mature EEA1 positive endosomes cluster perinuclearly, wherein signal is continuously activated until degradation.^30^, ^39^, ^40^ However, EEA1 showed scattered patterns in LY6K‐depleted cells (Figure 5D), possibly due to impaired clathrin‐mediated endocytosis and decreased clathrin levels (Figure 5A,C). Although how LY6K depletion results in lower clathrin levels remains ambiguous. Additionally, clathrin‐coated pits and vesicles and CAV‐1‐coated vesicles were barely observed in LY6K‐depleted cells. Accordingly, LY6K depletion affects from early stages of membrane invagination to formation of clathrin‐coated vesicles.

In conclusion, LY6K contributes to cervical cancer metastasis by regulating TGF‐β and EGF receptors and their signaling pathways. Since TGF‐β and EGF signals regulate tumor initiation and metastasis,^41^, ^42^ elucidating LY6K expression during cancer progression is important. Additionally, since high plasma membrane‐localized expression level of LY6K is observed in cervical cancer patients, it may be therapeutically targeted by chimeric antigen receptor‐NK and T cells.

## AUTHOR CONTRIBUTIONS

**Sujeong Park:** Data curation (equal); formal analysis (equal); investigation (equal); methodology (equal); resources (equal); validation (equal); visualization (equal); writing – original draft (equal); writing – review and editing (equal). **Doyeon Park:** Data curation (equal); formal analysis (equal); investigation (equal); methodology (equal); visualization (equal). **Sora Han:** Funding acquisition (equal); methodology (equal); project administration (equal); supervision (equal); writing – review and editing (equal). **Ga Eun Chung:** Data curation (equal); formal analysis (equal); investigation (equal); validation (equal). **Sujung Soh:** Methodology (equal); resources (equal); writing – review and editing (equal). **Hye In Ka:** Funding acquisition (equal); methodology (equal); resources (equal); writing – review and editing (equal). **Hyun Jeong Joo:** Methodology (equal); resources (equal); writing – review and editing (equal). **Young Yang:** Conceptualization (equal); data curation (equal); funding acquisition (equal); methodology (equal); project administration (equal); supervision (equal); writing – original draft (equal); writing – review and editing (equal).

## FUNDING INFORMATION

This research was kindly supported by Basic Science Research Program through the National Research Foundation of Korea (NRF) funded by the Ministry of Education (2021R1A2C3003414, 2021R1A6A1A03038890) and Korea Basic Science Institute (National Research Facilities and Equipment Center) grant funded by the Ministry of Education (2021R1A6C101A564).

## CONFLICT OF INTEREST STATEMENT

The authors declare that there are no conflicts of interest.

## ETHICS STATEMENT

Not applicable.

## PATIENT APPROVAL STATEMENT

Not applicable.

## CONSENT FOR PUBLICATION

All authors have consented for the publication.

## Supporting information


Appendix S1.

## Data Availability

All data used or analyzed during this study are included in this article.
