# Supplementary material for: LY6K depletion modulates TGF‐β and EGF signaling
Source: Cancer Med. 2023 Apr 19;12(11):12593–607. doi: 10.1002/cam4.5940 (PMC10278532; doi:10.1002/cam4.5940)
Supplement: Supplementary file 1 — Appendix S1. [file CAM4-12-12593-s001.zip › cam45940-sup-0001-supinfo.docx]

**Figure S1**. Immunoblotting analysis of TGF-β and EGF signaling pathways after siLY6K#3 transfection in HeLa and SiHa cells. Cells transfected with 20 ng/L of siLY6K#3 for 48 h were incubated with 5 ng/mL of TGF-β and 10 ng/mL of EGF for 1 h and 24 h. The intensities of p-EGFR, p-AKT, p-ERK, and p-Smad2 bands were quantified with ImageJ software.

**Figure S2**. Raw data of immunoblotting (A) Full blot images of Figure 1C (B) Full blot images of Figure 2A

**Figure S3**. Raw data of immunoblotting (A) Full blot images of Figure 3A.

**Figure S4**. Raw data of immunoblotting (A) Full blot images of Figure 3B.

**Figure S5**. Raw data of immunoblotting (A) Full blot images of Figure 3C (B) Full blot images of Figure 3D (C) Full blot images of Figure 3E.

**Figure S6**. Raw data of immunoblotting (A) Full blot images of Figure 4A.

**Figure S7**. Raw data of immunoblotting (A) Full blot images of Figure 5A.

**Figure S8**. Raw data of immunoblotting (A) Full blot images of Figure S1.
