# Supplementary figures and images for: LY6K depletion modulates TGF‐β and EGF signaling
Source: Cancer Med. 2023 Apr 19;12(11):12593–607. doi: 10.1002/cam4.5940 (PMC10278532; doi:10.1002/cam4.5940)

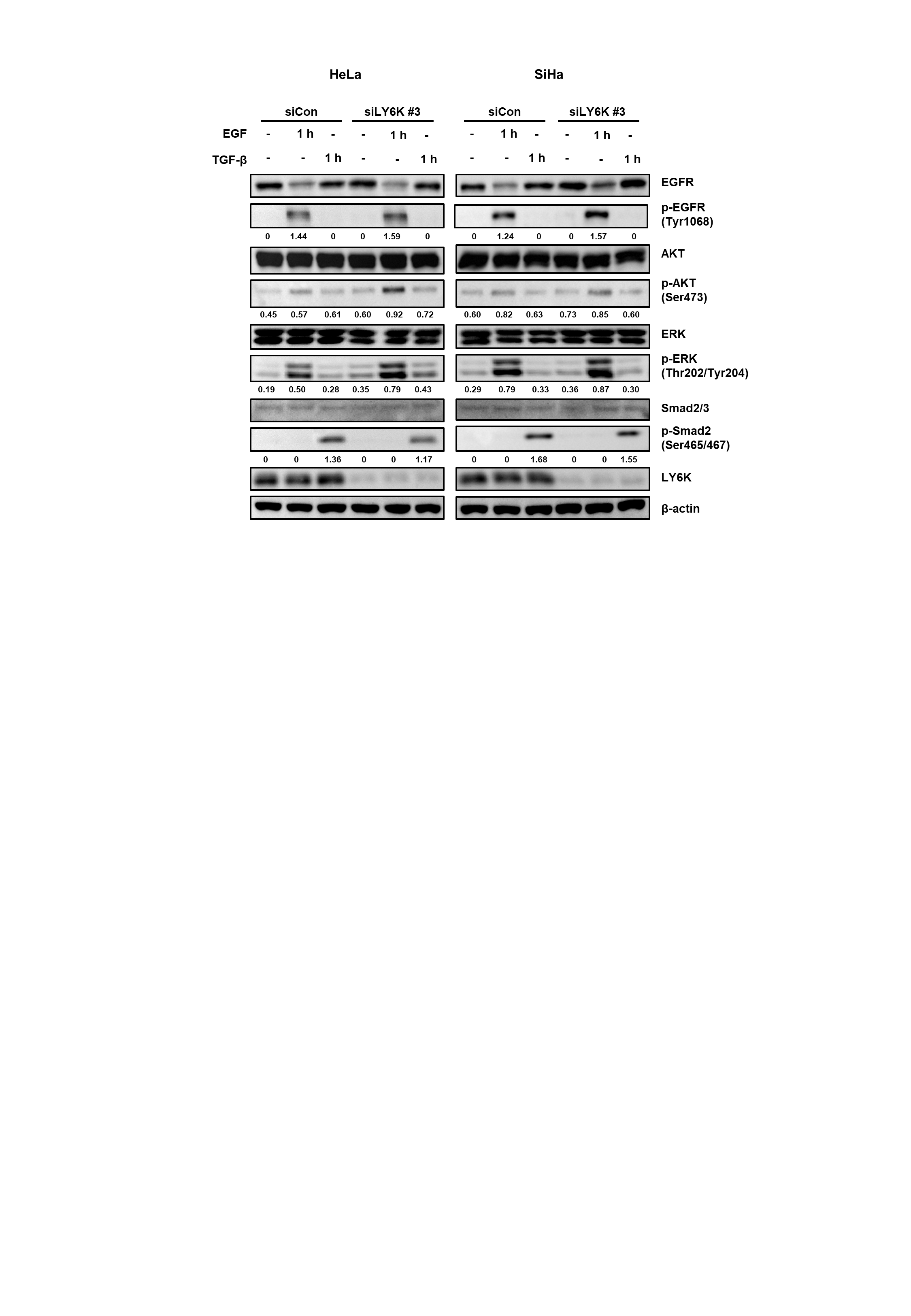

Supplement: Supplementary file 1 — Appendix S1. [file CAM4-12-12593-s001.zip › cam45940-sup-0001-FigureS1.TIF]

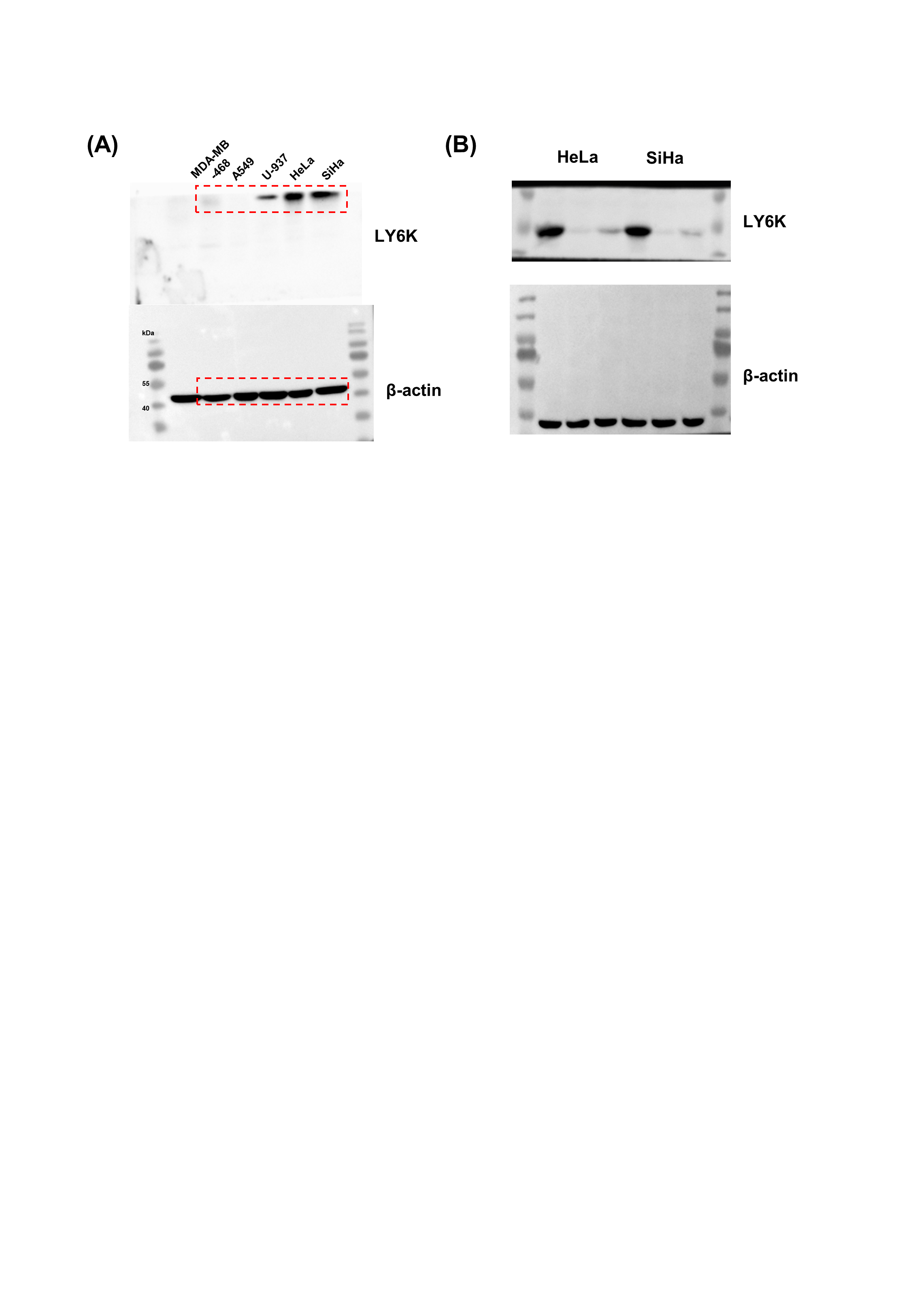

Supplement: Supplementary file 1 — Appendix S1. [file CAM4-12-12593-s001.zip › cam45940-sup-0002-FigureS2.TIF]

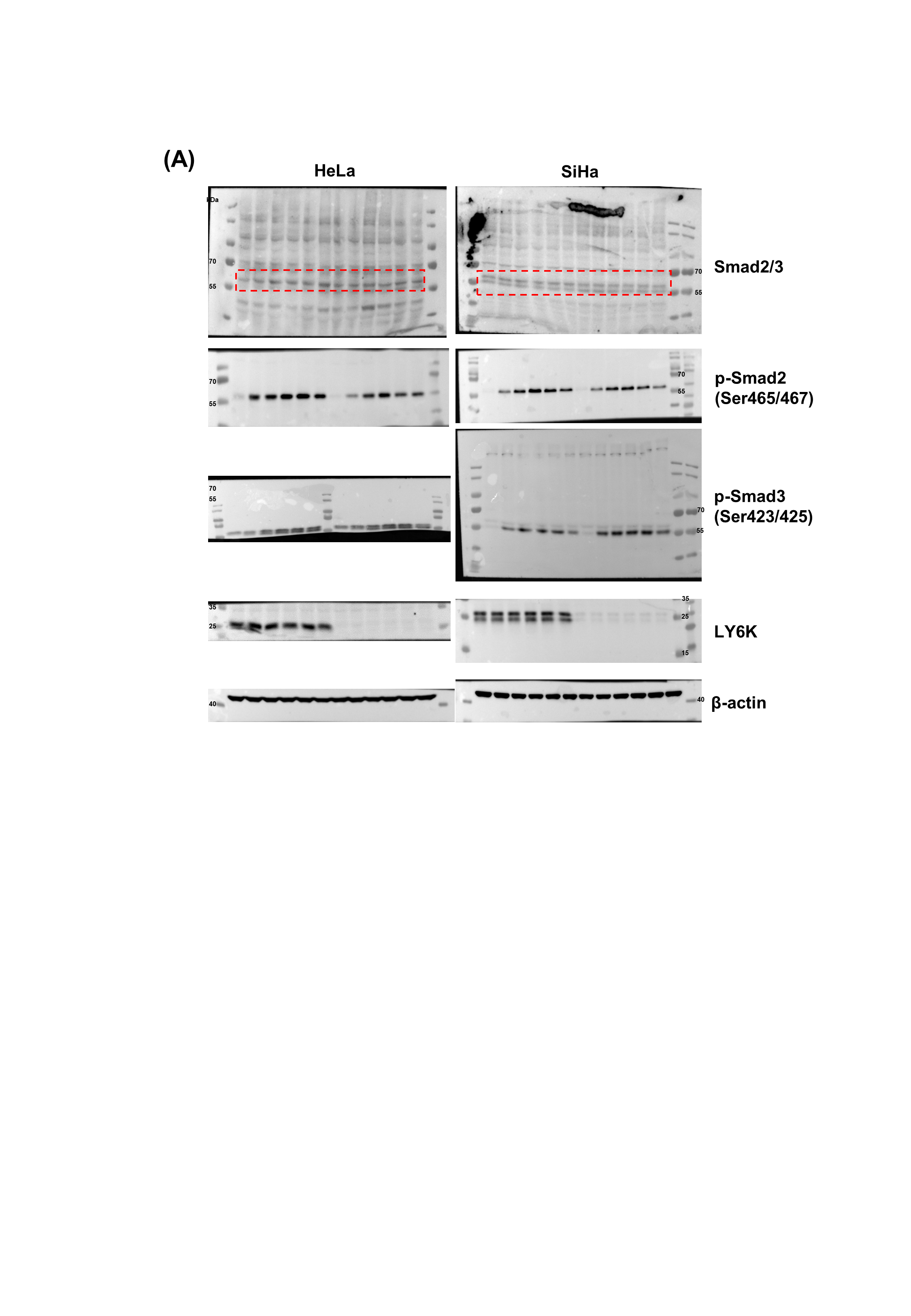

Supplement: Supplementary file 1 — Appendix S1. [file CAM4-12-12593-s001.zip › cam45940-sup-0003-FigureS3.TIF]

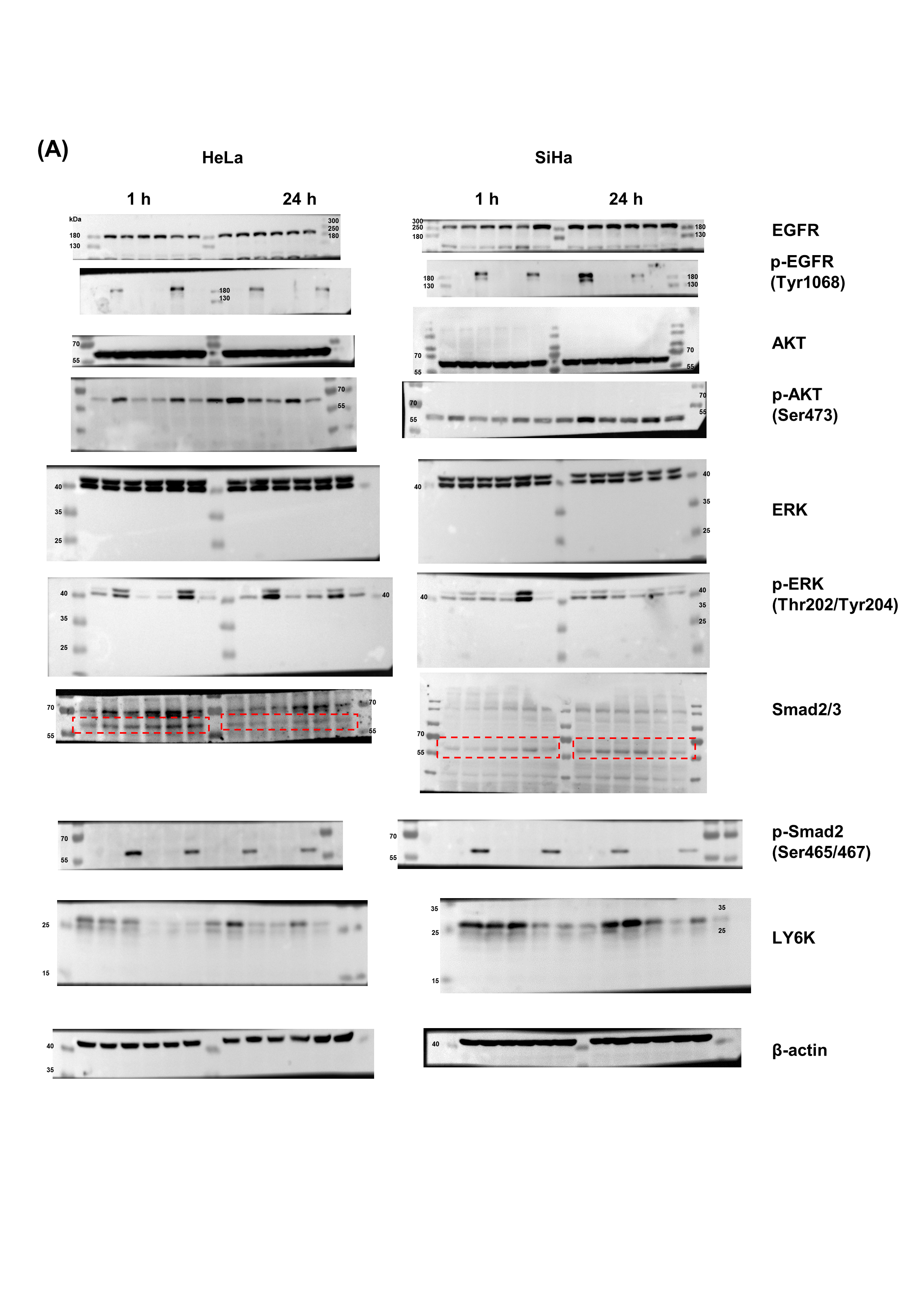

Supplement: Supplementary file 1 — Appendix S1. [file CAM4-12-12593-s001.zip › cam45940-sup-0004-FigureS4.TIF]

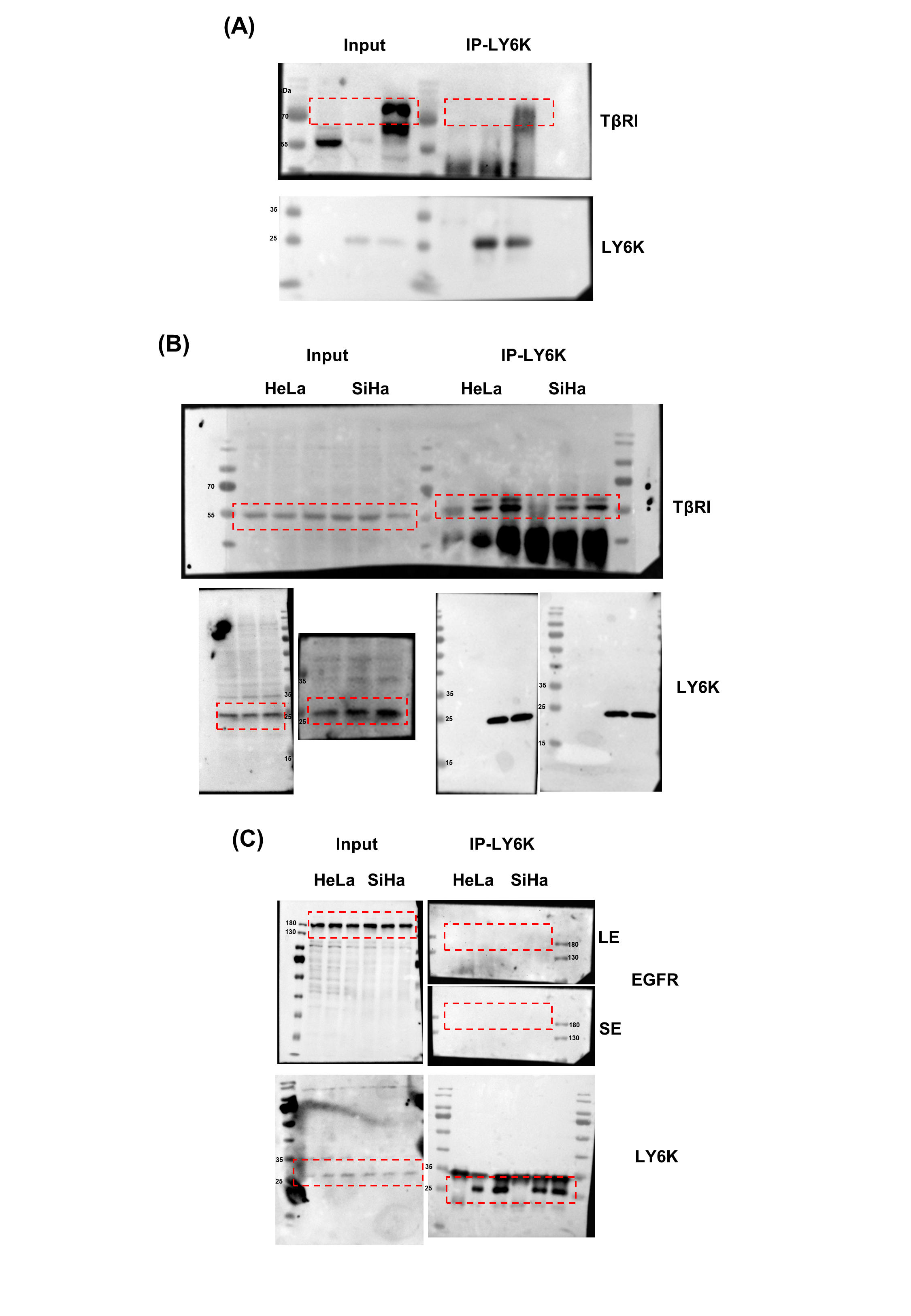

Supplement: Supplementary file 1 — Appendix S1. [file CAM4-12-12593-s001.zip › cam45940-sup-0005-FigureS5.TIF]

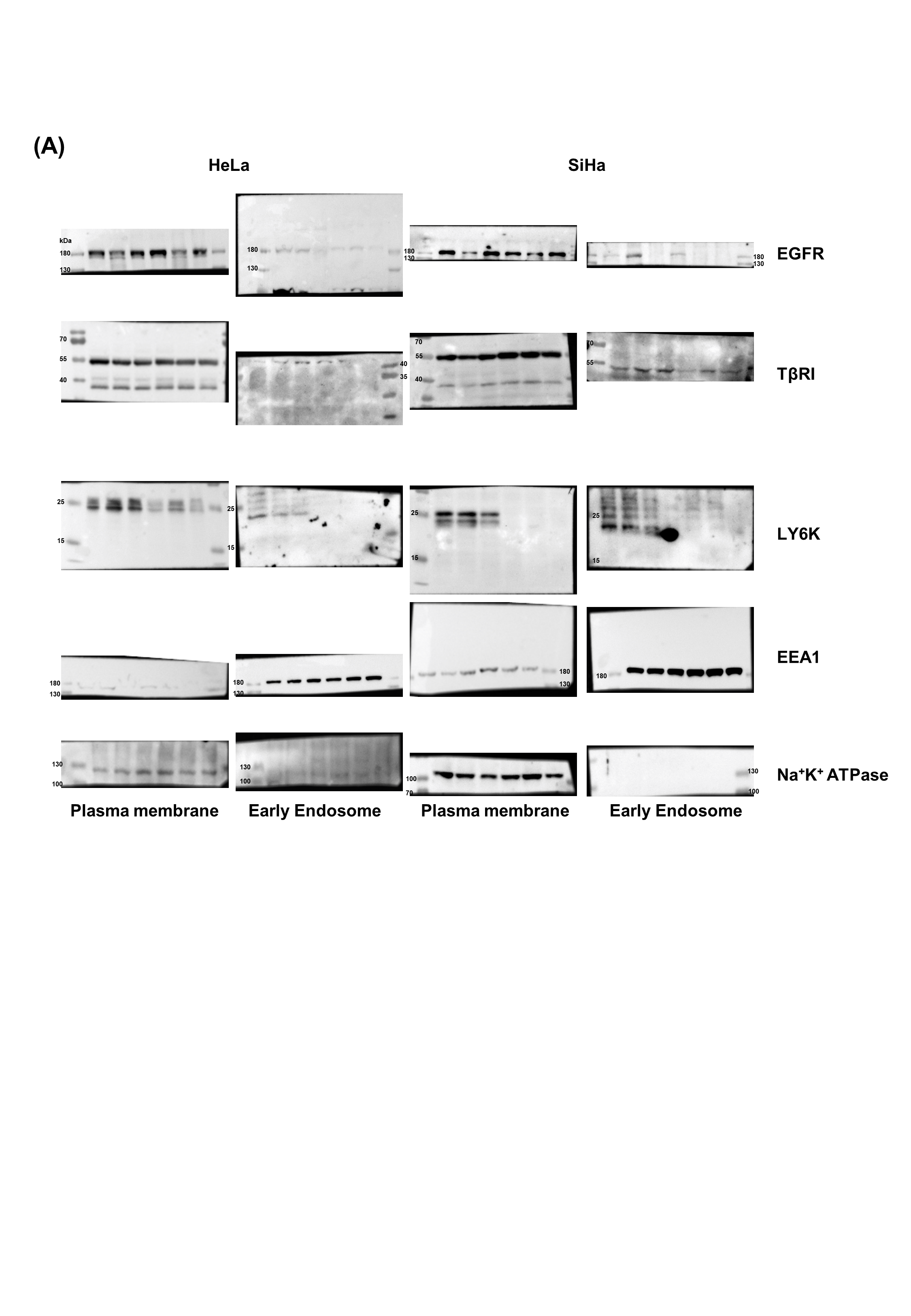

Supplement: Supplementary file 1 — Appendix S1. [file CAM4-12-12593-s001.zip › cam45940-sup-0006-FigureS6.TIF]

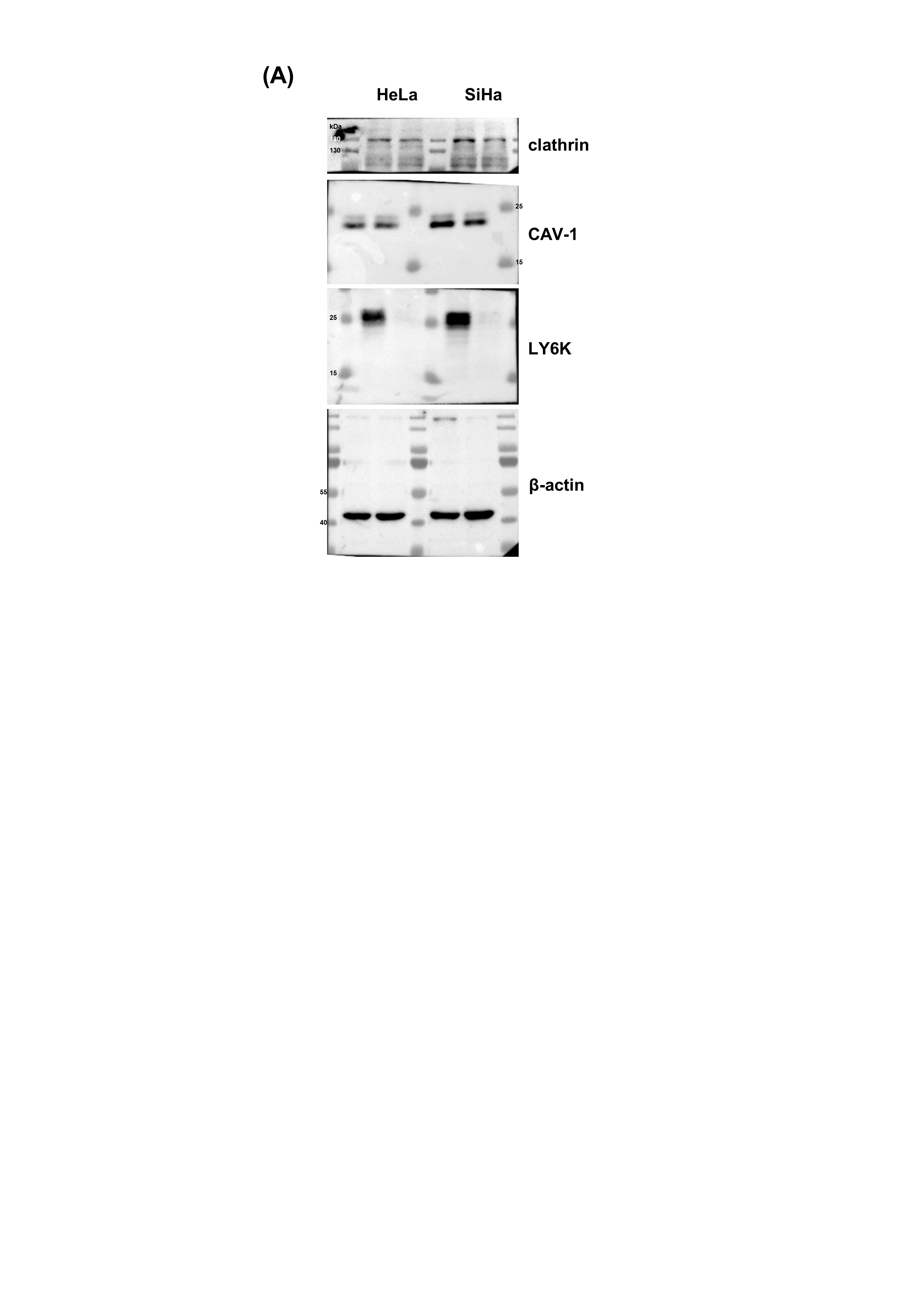

Supplement: Supplementary file 1 — Appendix S1. [file CAM4-12-12593-s001.zip › cam45940-sup-0007-FigureS7.TIF]

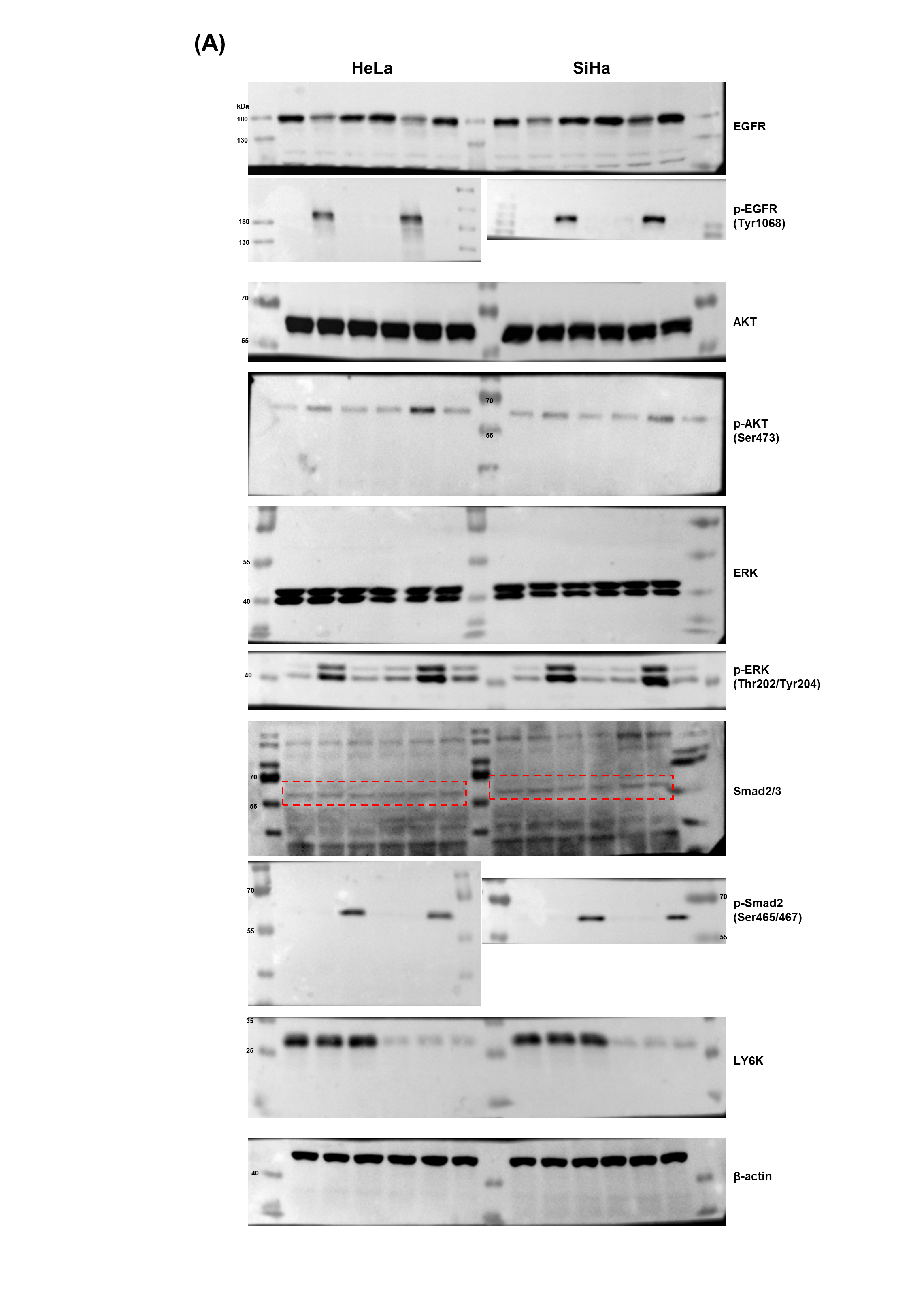

Supplement: Supplementary file 1 — Appendix S1. [file CAM4-12-12593-s001.zip › cam45940-sup-0008-FigureS8.TIF]
